# Inhibitory Effect of Puroindoline Peptides on *Campylobacter jejuni* Growth and Biofilm Formation

**DOI:** 10.3389/fmicb.2021.702762

**Published:** 2021-07-02

**Authors:** Prabhat K. Talukdar, Kyrah L. Turner, Torin M. Crockett, Xiaonan Lu, Craig F. Morris, Michael E. Konkel

**Affiliations:** ^1^School of Molecular Biosciences, College of Veterinary Medicine, Washington State University, Pullman, WA, United States; ^2^Department of Food Science and Agricultural Chemistry, Faculty of Agricultural and Environmental Sciences, McGill University, Montréal, QC, Canada; ^3^Western Wheat Quality Lab, U.S. Department of Agriculture–Agricultural Research Service, Pullman, WA, United States

**Keywords:** PinA, PinB, antimicrobial agent, foodborne pathogen, indoline

## Abstract

Puroindolines are small, amphipathic, wheat proteins that determine the hardness of the wheat kernel and protect crops from different pathogens. Puroindoline A (PinA) and puroindoline B (PinB) are two major isoforms of puroindolines. These proteins have antibacterial and antifungal properties mainly attributed to their characteristic tryptophan-rich domains (TRDs). In this *in vitro* study, we investigated the antimicrobial effect of PinA and PinB synthetic peptides against the growth and biofilm formation of *Campylobacter jejuni*. *C. jejuni* is an important microaerobic, foodborne pathogen that causes gastrointestinal and neurological diseases in humans. Our results showed that: (1) PinA, but not PinB, has strong antimicrobial activity against *C. jejuni* clinical strains 81-176 and F38011, *Escherichia coli* O157:H7, methicillin-resistant *Staphylococcus aureus*, *Salmonella enterica* serovar Typhimurium, and *Listeria monocytogenes*; (2) The substitution of two tryptophan residues to glycine (W→G) in the TRD of PinA abolishes its antimicrobial activity against these microorganisms; (3) PinA functions additively with two common antibiotics (ciprofloxacin and erythromycin) to inhibit or inactivate *C. jejuni* strains; (4) PinA damages the *C. jejuni* cellular membrane, (5) PinA is cytotoxic to human INT 407 cells at high concentrations; and (6) PinA inhibits *C. jejuni* biofilm formation. In summary, this study demonstrates the antimicrobial activity of PinA against *C. jejuni* growth and biofilm formation and further confirms the potential use of PinA as a therapeutic agent in health care or as preservatives in the agri-food industry.

## Introduction

Microorganisms, including commensals and pathogens, have developed antimicrobial resistance to existing drugs (Prestinaci et al., 2015; Nji et al., 2021). Therefore, the development of alternative therapies is warranted to combat the emergence and spread of multi-drug resistant bacteria. Antimicrobial peptides (AMPs) from natural sources such as plants, insects, and other organisms are considered as potential alternatives to conventional antibiotics due to their broad-spectrum antimicrobial activities and the low chance of microbial resistance development (Tam et al., 2015; Lei et al., 2019). Natural AMPs are part of the innate immunity of plants, animals, and other organisms and protect the host by rapidly killing invading organisms (Diamond et al., 2009). AMPs are generally low molecular weight proteins, have globular structures with disulfide bonds, and contain cysteine or tryptophan-rich domains (TRDs) (Bahar and Ren, 2013). Certain cationic AMPs have high affinity to the negatively charged microbial lipid membrane whereas low affinity to the eukaryotic membrane (Hollmann et al., 2018). AMPs differ in their secondary and tertiary structures and amino acid sequences. Based on the type of AMP and the target organism, AMPs confer two distinct antimicrobial actions. Some AMPs cause cell death by cell lysis or disrupt membranes by forming ion channels or pores without lysis (Li et al., 2017; Raheem and Straus, 2019). Other AMPs inactivate cells by disrupting intracellular targets such as DNA, RNA, or proteins (Le et al., 2017). AMPs apply either mode of action simultaneously and independently or utilize a single mode of action, either membrane disruption or intracellular component damage. In general, bacteria are more prone to the effect of AMPs from natural sources than to synthetic drugs, as AMPs often act non-specifically on one or more target(s) that the bacteria cannot restore; e.g., most bacteria are unable to restore the damage to the cell membrane caused by AMPs (Cole and Nizet, 2016). Therefore, the search for AMPs from natural sources as well as acquiring information on the antimicrobial activities of already known AMPs against a wider range of organisms is crucial to finding suitable natural alternatives to existing drugs.

Plants have natural defense mechanisms to protect them from physical, chemical, and biological stresses. For example, cysteine and tryptophan-rich peptides are responsible for protecting plants from the attack of bacteria, fungi, and viruses (Nawrot et al., 2014; Campos et al., 2018). In addition, plant seeds contain many proteins with potential antimicrobial activities (Tam et al., 2015). Puroindolines are present throughout the wheat (*Triticum aestivum*) endosperm. These small (13 kDa), amphipathic proteins are unique due to their dual role as a determinant of wheat quality and the protector of crops from different diseases. Puroindolines determine the hardness of the wheat kernel and, therefore, the milling and baking quality (Giroux and Morris, 1998; Morris, 2002; Giroux et al., 2003). Puroindoline A (PinA) and puroindoline B (PinB) are the two major isoforms of puroindolines and share 60% sequence identity at the amino acid level and 70% identity at the nucleic acid level. Both proteins are folded by five disulfide bonds, contain ten highly conserved cysteine residues, and a characteristic TRD with five tryptophan residues in PinA (WRWWKWWK) and three tryptophan residues in PinB (WPTKWWK) (Blochet et al., 1993; Gautier et al., 1994; Morris, 2019). Both functional PinA and PinB are required to maintain the softness of the grain texture, while the absence or mutation (amino acid substitution) in either one of the proteins results in hard wheat kernels (Giroux and Morris, 1998; Morris, 2002; Giroux et al., 2003).

Another important role of puroindolines is to enhance the disease-resistance of crops against various pathogens, including bacteria and fungi (Krishnamurthy et al., 2001; Giroux et al., 2003; Morris, 2019). *In vitro* studies with PinA and PinB full proteins or shorter peptides demonstrate that these proteins or peptides have antimicrobial activity against some common Gram-positive and Gram-negative bacteria, including *Escherichia coli*, *Staphylococcus aureus*, *Staphylococcus epidermidis*, *Bacillus subtilis*, *Listeria monocytogenes*, and a few fungi, including *Candida albicans* and *Aspergillus flavus* (Charnet et al., 2003; Jing et al., 2003; Capparelli et al., 2005; Palumbo et al., 2010; Miao et al., 2012; Alfred et al., 2013; Haney et al., 2013; Lv et al., 2019; Tian et al., 2021). A gene expression analysis also showed that the PinA gene is induced when rice is attacked by a pathogen or wounded (Evrard et al., 2007). The antimicrobial activity of PinA and PinB is mainly attributed to the presence of TRDs in their structure. TRD-rich peptides have a high affinity to the lipid of the negatively charged bacterial membrane. The TRD region itself, when cloned into a recombinant vector, possesses an inhibitory effect similar to the full protein (Capparelli et al., 2006; Capparelli et al., 2007). The mode of action of PinA is different from PinB. PinA is the membrane destabilizing peptide, forming pores in lipid bilayers in bacterial membranes (Krishnamurthy et al., 2001; Charnet et al., 2003; Jing et al., 2003). In contrast, PinB targets an intracellular component. The primary mode of action of the PinB peptide involves the binding to DNA and inhibiting DNA replication (Bhave and Morris, 2008; Alfred et al., 2013).

*Campylobacter jejuni* is a Gram-negative, curved-shaped, motile, microaerophilic bacterium and one of the leading bacterial causes of foodborne illnesses and gastrointestinal diseases in the world (Kaakoush et al., 2015). The symptoms of *C. jejuni* infection, commonly known as campylobacteriosis, include diarrhea, fever, and abdominal cramping. Some strains of *C. jejuni* also cause Guillain–Barré syndrome (GBS), a form of flaccid paralysis (Nyati and Nyati, 2013). Chickens are the natural reservoir of *C. jejuni* and the main source of *C. jejuni* infection in humans. The use of antibiotics in poultry production is one of the major factors for the development of antibiotic resistance in *C. jejuni* (Sahin et al., 2002; Skarp et al., 2016). Antibiotic-resistant *C. jejuni* isolates have been observed and reported in recent years (Marotta et al., 2020; Tang et al., 2020; Hull et al., 2021). A recent study reported that some natural products, including plant extracts, essential oils, and pure phytochemicals, are effective against drug-sensitive and drug-resistant *Campylobacter* strains (Gahamanyi et al., 2020). These natural products can be used alone or in combination to kill *C. jejuni*. The findings of new natural products, such as puroindolines, could be beneficial to minimize *C. jejuni* growth and biofilm formation in the poultry industry and food processing facilities.

Based on the potential antimicrobial activity of PinA and PinB peptides against a few common Gram-positive and Gram-negative bacteria (Capparelli et al., 2005; Palumbo et al., 2010; Miao et al., 2012; Lv et al., 2019), it is imperative to know whether these proteins can inhibit a broader range of organisms, especially against major foodborne pathogens. This information is key to the application of puroindoline proteins or peptides as therapeutics, preservatives, or preventive drugs. However, to date, no information on the effect of puroindolines against *C. jejuni* and other major foodborne pathogen has been reported. In this study, we used PinA and PinB synthetic peptides that harbor the wild-type and a mutant copy of TRDs independently or in combination with other existing antibiotics and determined the efficacy of PinA and PinB peptides on *C. jejuni* growth and biofilm formation. In addition, we assessed the potential mechanism of action of PinA peptides on *C. jejuni* as well as the cytotoxic effect of PinA and PinB peptides on one human epithelial cell line as well as sheep red blood cells (SRBCs). In summary, we identified that the PinA peptide demonstrates an antimicrobial effect on *C. jejuni* growth and biofilm formation and, therefore, can be used as a potential therapeutic agent to treat *C. jejuni* infection.

## Materials and Methods

### Bacterial Strains, Host Cells, and Growth Conditions

*C. jejuni* clinical strains 81-176 and F38011 were cultured every 24 to 48 h on Mueller-Hinton (MH) agar containing 5% citrated bovine blood (MH-blood agar) or in MH broth in a shaking orbital incubator with microaerobic conditions (5% O_2_, 10% CO_2_, 85% N_2_) at 37°C. The generic isolates of *E. coli* O157:H7, *L. monocytogenes*, and *Salmonella enterica* serovar Typhimurium (*S.* Typhimurium) were cultured on LB agar plates in an aerobic chamber at 37°C, and methicillin-resistant *S. aureus* (MRSA) were cultured on an MH-blood agar plate in an aerobic chamber at 37°C.

The toxicity of the puroindoline peptides was assessed using INT 407 epithelial cells (ATCC CCL-6) and SRBCs, as described below. The INT 407 cells (a derivative of HeLa cells) were cultured in Minimal Essential Medium (MEM) (Gibco, Grand Island, NY, United States) supplemented with 10% fetal bovine serum (FBS) and 1 mM sodium pyruvate at 37°C in humidified condition (5% CO_2_).

### Synthesis of Peptides and Preparation of Antimicrobial Substances

Four different puroindoline peptides, each containing 18 amino acids and one TRD, were synthesized by Genemed Synthesis Inc. (San Antonio, TX, United States). The names and sequences of the peptides are the following: PinA WT, TMKDFPVTWRW**W**KW**W**KGG; PinA mutant, TMKDFPVTWRW**G**KW**G**KGG; PinB WT, TMKDFPVTWPT**K**WW**K**GG; and PinB mutant, TMKDFPVTWPT**G**WW**G**GG. PinA mutant peptide was obtained by replacing two tryptophan residues with two glycine residues at positions 12 and 15 (bold, above). To mutate the PinB peptide, two glycine residues were incorporated in place of two lysine residues at positions 12 and 15. In addition, two synthetic peptides containing PinA and PinB with a glycine-rich linker sequence (bold, below) were synthesized. The sequences of the two linker peptides are the following: PinA-linker-PinB, TMKDFPVTWRWWKWWKGG**GGSGG**TMKDFPVTWPTKW WKGG; and PinB-linker-PinA, TMKDFPVTWPTKWWK GG**GGSGG**TMKDFPVTWRWWKWWKGG. The peptides were synthesized and shipped as dry lyophilized powders. Upon receipt, peptides were stored at −20°C or suspended in dimethyl sulfoxide (DMSO) at a concentration of 50 mg/mL for further use. Stock solutions of erythromycin (25 mg/mL) and ciprofloxacin (10 mg/mL) were prepared in deionized water and sterilized by filtration.

### Antimicrobial Susceptibility Test

To determine the minimum inhibitory concentration (MIC) and minimum bactericidal concentration (MBC) of the puroindoline peptides, ciprofloxacin, and erythromycin, antimicrobial susceptibility tests were performed using the broth microdilution technique according to the Clinical and Laboratory Standards Institute (CLSI) guidelines (Clinical and Laboratory Standards Institute, 2018). Briefly, 100 μL of 2× concentration of MH broth was added in each well of a sterile round-bottom 96-well plate, and 100 μL of the peptides (PinA, PinB, PinA mutant, PinB mutant, PinA-linker-PinB, PinB-linker-PinA, and PinA+PinB) or the antibiotics (ciprofloxacin and erythromycin) was added to the first column of wells on the 96-well plate. Two-fold serial dilutions were made. The bacterial cultures were grown overnight in MH broth under the appropriate conditions, pelleted by centrifugation, and suspended in MH broth at 5 × 10^6^ CFU/mL. Ten microliters of bacterial suspension (∼5 × 10^4^ bacteria) was used to inoculate every well except the media control wells in 96-well microtiter plate. The plate was incubated in an orbital shaker at 37°C in a microaerobic condition for 48 h for *C. jejuni* strains or in aerobic conditions for 24 h for other bacteria. After incubation, the optical density was determined using a 96-well plate reader (BioTek Instruments Inc., Winooski, VT, United States) at 595 nm (OD_595_). MIC is defined as the lowest concentration of the antimicrobial agent that results in the absence of visible growth. The dose-response curves were created by log-transforming the OD_595_ readings.

To determine the MBC, a sterile 96-well replicator tool was used to spot each well from the 96-well plate onto a 100 mm^2^ MH agar plate. The plate was allowed to incubate for 24–48 h under conditions specific to the bacteria. The MBC was determined by the lowest concentration of the antimicrobial agent that resulted in no bacterial growth (>99.9% reduction) or clear spot on the agar plate.

### Isobologram Analysis

The interaction between peptide and antibiotics was determined using the checkerboard assay and subsequent isobologram analysis as described previously (Hakeem et al., 2019). Briefly, defined combinations of each antimicrobial agent (i.e., IC_50_ of PinA and IC_50_ of ciprofloxacin) were added to the wells of a 96-well plate. The following equation was used to calculate the IC amounts:

$$I\,C_{F}={\left(\frac{F}{100-F}\right)}^{\frac{1}{H}}\times I\,C_{50}$$

where F represents the percentage of reduction, H represents the Hill slope from the dose-response curves, and IC_50_ is the concentration that gives a 50% reduction in bacterial growth.

Two different serial dilutions were made for the checkerboard assay. A two-fold serial dilution was made for one antimicrobial agent from left to right in a 96-well plate. Another two-fold serial dilution was made for the second antimicrobial agent from top to bottom in the same plate. Then, *C. jejuni* culture was added into each well at a density of 5 × 10^4^ bacteria/well. The plate was incubated with orbital shaking at 220 rpm for 48 h, and growth was determined using a microplate reader at 595 nm (OD_595_). The IC_50_ of the two antimicrobial substances were plotted on the axes, and the additive line connecting the two IC_50_s was drawn. The combination of antimicrobial agents (axial point) that produced a 50% reduction in bacterial growth was plotted on the graph. An axial point landing on the additive line indicates an additive effect, under the additive line indicates a synergistic effect, and above the additive line indicates an antagonistic effect.

### Ethidium Bromide Accumulation Assay

The ethidium bromide accumulation assay was performed as described elsewhere with minor modifications (Lin et al., 2002). Overnight broth cultures of *C. jejuni* strain 81-176 and strain F38011 were pelleted and resuspended in 1× phosphate-buffered saline (PBS) to an OD_540_ of 0.5–0.6. Each bacterial suspension was added to a 96-well plate, and various concentrations of the peptides were added to four replicate wells. As a positive control, 1% Triton X-100 and 1% SDS were added to separate wells. After 60 min of incubation at 37°C, ethidium bromide was added to a final concentration of 2 μg/mL. The plate was read kinetically on a VICTOR X5 multilabel plate reader (PerkinElmer, Waltham, MA, United States) using an excitation wavelength of 530 nm and an emission wavelength of 610 nm, taking measurements every 2 min.

### Determination of Cytosolic ATP

Cytosolic ATP of *C. jejuni* cultures was determined using the ATP Bioluminescence Assay Kit (Roche Diagnostics, Mannheim, Germany) according to the manufacturer’s recommendations. Overnight bacteria culture of *C. jejuni* strain 81-176 was pelleted and resuspended with 1× PBS to an OD_540_ of 0.2. Bacteria were added to a 96-well plate at a density of 5 × 10^6^ CFU/well. The peptides were added to the well at different concentrations, and the plate was incubated at 37°C in a microaerobic condition. Fifty microliters of the bacterial cultures were removed at different time points and transferred to a new 96-well black plate, which was kept on ice until measurement. Luciferase reagent (50 μL) provided with the ATP assay kit was added, and the bioluminescence activity was immediately recorded using a VICTOR X5 Multilabel Plate Reader (PerkinElmer) at 20°C. The amount of cytosolic ATP was measured by taking the value as a relative fluorescence unit (RLU).

### Lactate Dehydrogenase Assay

The cytotoxicity of puroindoline peptides on INT 407 cells was monitored using the lactate dehydrogenase (LDH) activity assay kit (Millipore Sigma, Danvers, MA, United States) following the manufacturer’s guidelines. LDH is released from damaged cells into the medium. Briefly, INT 407 cells were seeded into a 96 well plate at a density of 2.5 × 10^4^ cells/well in 200 μL growth media (MEM supplemented by 10% FBS) and incubated for 24 h at 37°C in humidified (5% CO_2_) conditions. The next day, cells were rinsed twice with MEM without FBS, and different concentrations of peptides were added. Cells were incubated for 3 h, 6 h or 24 h at 37°C. As a positive control, 0.1% Triton X-100 was used. Media only was used as a negative control. In the presence of Triton X-100, cells release maximum LDH. After the appropriate incubation point, the tissue culture plate was centrifuged at 2000 rpm for 5 min. Ten microliters of supernatant from each well was transferred to a new 96-well plate, and 100 μL of reconstituted LDH substrate mixed with LDH buffer provided with the kit was added to each well. The plate was kept for 30 min in a dark room at 37°C. Stop solution was then added to each well and left for 1 h. Absorbance was recorded at 450 nm (OD_450_) on a BioTek 96-well plate reader.

### MTT Assay

The viability of INT 407 cells was assessed by MTT assay according to the manufacturer’s recommendations (Roche Diagnostics, Mannheim, Germany). Briefly, INT 407 cells were seeded into a 96-well plate at a density of 2.5 × 10^4^ cells/well in 200 μL of growth media (MEM supplemented by 10% FBS) and incubated for 24 h at 37°C under humidified conditions. Cells were rinsed twice with MEM without FBS, and different concentrations of peptides were added into the well. Triton X-100 was added as a positive control and growth medium only was added as a negative control. After treatment, the cells were incubated for different time points (3, 6, or 24 h) at 37°C in a humidified condition. At the end of each experiment, 100 μL of culture medium was removed into a new 96-well plate, and 25 μL of MTT stock solution was added into each well. The plates were incubated for 4 h at 37°C. Then, 100 μL of the solubilizing solution was added to each well, and the plate was incubated overnight at 37°C. After cooling, the plates were read at 570 nm (OD_570_) on a BioTek 96-well plate reader.

### Hemolysis Assay

The potential toxicity of the peptides was determined using a hemolysis assay. Briefly, SRBCs (Hardy Diagnostics, Santa Monica, CA, United States) were washed three times with 1× PBS. Aliquots containing approximately 10^8^ cells/mL were incubated with different concentrations of the peptides for 60 min at 37°C in a 96-well tray. The plates were then centrifuged, and the absorbance of each supernatant was measured at 540 nm. Zero hemolysis (blank) and 100% hemolysis were determined in phosphate buffer and 1% Triton X-100, respectively.

### Biofilm Formation and Crystal Violet Staining

An overnight culture of *C. jejuni* was diluted to an OD_540_ of 0.03, 0.1, 0.5, and 1.0 in MH broth. A total of 200 μL of the diluted bacterial culture was added into each well of a 96-well tray and incubated in a microaerobic chamber (85% N_2_, 10% CO_2_, and 5% O_2_) at 37°C for 72 h without agitation. Based on the results of preliminary experiments, all subsequent experiments were performed using an OD_540_ of 0.1. To test the inhibitory effect of antimicrobial agents, a 2× concentration of peptides and/or antibiotics was added to the 96-well tray. Bacterial cultures were then added at an OD_540_ of 0.1 in each well except for the media-only control wells.

Biofilm formation was determined by crystal violet staining. Briefly, the wells of the 96-well plate were rinsed with sterile deionized water and air-dried for 15 min. A solution of crystal violet [0.5% (w/v)] was added to each well and incubated for 15 min at room temperature to stain the biofilm. The wells were then rinsed with sterile deionized water and 95% ethanol (v/v) was added into each well and incubated for 10 min. The bound crystal violet was dissolved in 95% ethanol (v/v), and the amount of crystal violet was measured using a BioTek 96-well plate reader at 595 nm (OD_595_). As a negative control, a well incubated with MH broth was stained using the same method described above. The signal from the well incubated with the MH broth was subtracted from all values for background correction.

To determine the number of planktonic bacteria, the supernatants from the wells were transferred to a 1.5 mL centrifuge tube. The remaining biofilm associated with the well was washed twice with sterile 1× PBS and suspended with 200 μL 1× PBS. The serial dilution was performed and 100 μL of the suspension was plated on BHI agar. The plates were incubated at 37°C in a microaerobic condition for 48 h, and the colonies were counted. All experiments were done at least three times for reproducibility.

### Statistical Analysis

Statistical analysis was performed using GraphPad Prism 6.0g (La Jolla, CA, United States). The specific statistical tests are indicated in the figure legends.

## Results

### Antimicrobial Activity of PinA and PinB Peptides Against Gram-Positive and Gram-Negative Bacteria

The antimicrobial activity of PinA WT, PinB WT, PinA mutant, PinB mutant peptides against *C. jejuni* strains 81-176 and F38011, *E. coli* O157:H7, *S.* Typhimurium, MRSA, and *L. monocytogenes* were tested, and the MIC was determined (Table 1). PinA showed the strongest activity against the two *C. jejuni* strains with a MIC of 16 and 32 μg/mL for F38011 and 81-176, respectively. The lowest activity of PinA was observed for *S.* Typhimurium (MIC of 256 μg/mL). Similar assays were performed with the PinB peptide. However, PinB was identified to be ineffective against all the bacteria tested at a concentration of 512 μg/mL or lower. Similarly, no antimicrobial activity was observed with the PinA mutant and PinB mutant peptides. In addition, the antimicrobial activity of PinA-linker-PinB, PinB-linker-PinA, and the combination of PinA and PinB peptides together were tested against *C. jejuni* strains 81-176 and F38011 (Supplementary Figures 1A,B). Both PinA-linker-PinB and PinB-linker-PinA peptides showed very little inhibitory effect against *C. jejuni* strain 81-176. However, at 512 μg/mL, both peptides inhibited *C. jejuni* strain F38011. When PinA and PinB peptides were used at a 1:1 concentration, the inhibition was greater than the effect seen with two individual linker peptides.

**TABLE 1:** Minimum inhibitory concentration (MIC) of puroindoline peptides against Gram-positive and Gram-negative bacteria.

Organism	MIC (μg/mL)				MBC (μg/mL)
	
	PinA	PinB	PinA mutant	PinB mutant	PinA
*C. jejuni* 81-176	32	>512	>512	>512	64
*C. jejuni* F38011	16	>512	>512	>512	32
*E. coli* O157:H7	64	>512	>512	>512	256
*S.* Typhimurium	256	>512	>512	>512	512
MRSA	128	>512	>512	>512	256
*L. monocytogenes*	64	>512	>512	>512	64

**FIGURE 1:**
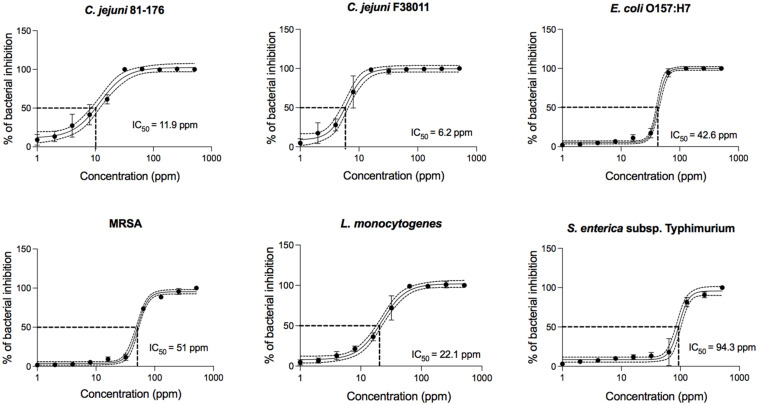
Concentration-effect curves for PinA against *C. jejuni* strain 81-176, *C. jejuni* strain F38011, *E. coli* O157:H7, *Salmonella enterica* subsp. *enterica* serovar Typhimurium (*Salmonella typhimurium*), methicillin-resistant *Staphylococcus aureus* (MRSA), and *Listeria monocytogenes*. The antimicrobial activity of PinA was determined by the broth micro-dilution method as described in section “Materials and Methods.” Bacterial cultures were treated with different concentrations of PinA and incubated for either 24 h (*E. coli* O157:H7, *S.* Typhimurium, MRSA, and *L. monocytogenes*) or 48 h (*C. jejuni* strains 81-176 and F38011) under appropriate conditions. The IC_50_ (50% of inactivated cells) was determined for each bacterium. Dashed lines indicate the PinA concentration that is required for a 50% reduction of the cells (IC_50_). The data represent a minimum of three biological replicates, and the error bars represent the average ± standard deviation.

To further define the antimicrobial activity of PinA against all bacteria tested in this study, concentration-effect curves were generated (Figure 1). Regardless of the bacterial isolate, PinA showed a *sigmoidal* concentration-effect curve. To compare the antimicrobial activity of PinA against the various bacteria tested, we determined the half-maximal inhibitory concentration (IC_50_), which is the value in the middle of the concentration-effect curve, or the concentration at which 50% of bacteria is inhibited. A lower IC_50_ indicates a greater antimicrobial activity (i.e., a greater potency). Ten concentration-effect points were tested to generate a concentration-effect curve for each bacterium. The negative control was each bacterium incubated in medium without the peptide. As expected, PinA exhibited the strongest activity against *C. jejuni* strain F38011 (IC_50_ = 6.2 ppm), followed by strain 81-176 (IC_50_ = 11.9 ppm), *L. monocytogenes* (IC_50_ = 22.1 ppm), *E. coli* O157:H7 (IC_50_ = 42.6 ppm), MRSA (IC_50_ = 51 ppm), and *S.* Typhimurium (IC_50_ = 94.3 ppm).

Finally, the IC_90_, IC_50_, and IC_10_ inhibitory concentration values were determined for the PinA WT peptide from the slope of the concentration-effect curve (Supplementary Table 1). The MBC of PinA ranged from 32 μg/mL for *C. jejuni* strain F38011 to 512 μg/mL for *S.* Typhimurium. Taken together, the PinA peptide was identified to have both bacteriostatic and bactericidal activity against both Gram-positive and Gram-negative bacteria.

### PinA Acts Additively With Erythromycin and Ciprofloxacin to Inhibit *C. jejuni* Growth

Based on the effectiveness of PinA against all Gram-positive and Gram-negative bacteria tested in this study and the fact that *C. jejuni* is a leading bacterial cause of foodborne illness worldwide, we shifted our focus to study the antimicrobial activity of PinA against two *C. jejuni* clinical strains (81-176 and F38011) and the potential of using PinA in combination with antibiotics commonly used to treat *C. jejuni* infections. Two antibiotics, erythromycin and ciprofloxacin, were selected to use in this study as these two antibiotics are being commonly used to treat individuals suffering from acute *C jejuni* infections.

**FIGURE 2:**
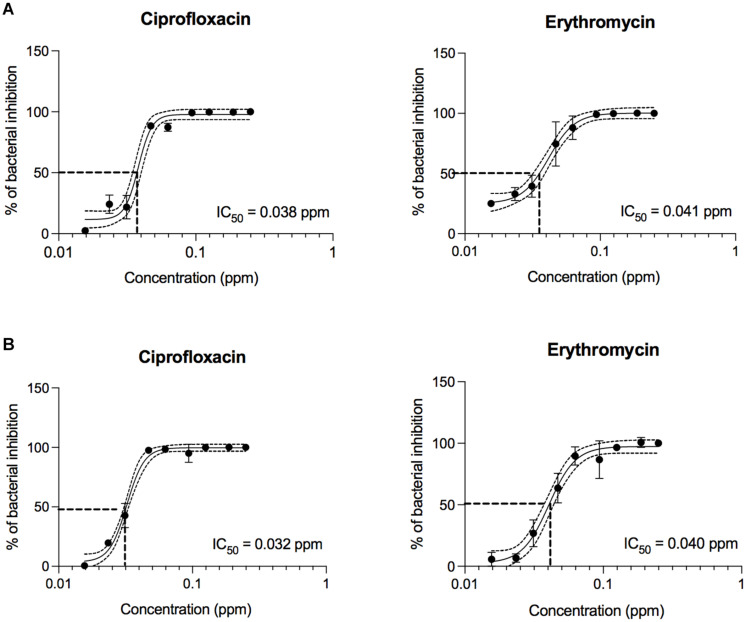
Concentration-effect curves for erythromycin and ciprofloxacin against *C. jejuni* strains. *C. jejuni* strains **(A)** 81-176 and **(B)** F38011 were treated with either erythromycin or ciprofloxacin and incubated for 48 h at 37°C in a microaerobic condition. The IC_50_ (50% of inactivated cells) was determined for each *C. jejuni* strain. Dashed lines indicate the antibiotic concentration that is required for a 50% reduction of the cells (IC_50_). The assay was repeated a minimum of three times to ensure reproducibility. Error bars represent the standard deviation from three separate assays.

Concentration-effect curves for erythromycin and ciprofloxacin were generated for *C. jejuni* clinical strains 81-176 and F38011. Reported are the IC_50_ values for the antibiotics against both *C. jejuni* clinical strains (Figures 2A,B) as well as the MIC, IC_90_, IC_50_, IC_10_, MBC values (Supplementary Table 2). The IC_50_ of ciprofloxacin (0.038 ppm and 0.032 ppm for strain 81-176 and F38011, respectively) and erythromycin (0.041 ppm and 0.040 ppm for strain 81-176 and F38011, respectively) was determined.

**FIGURE 3:**
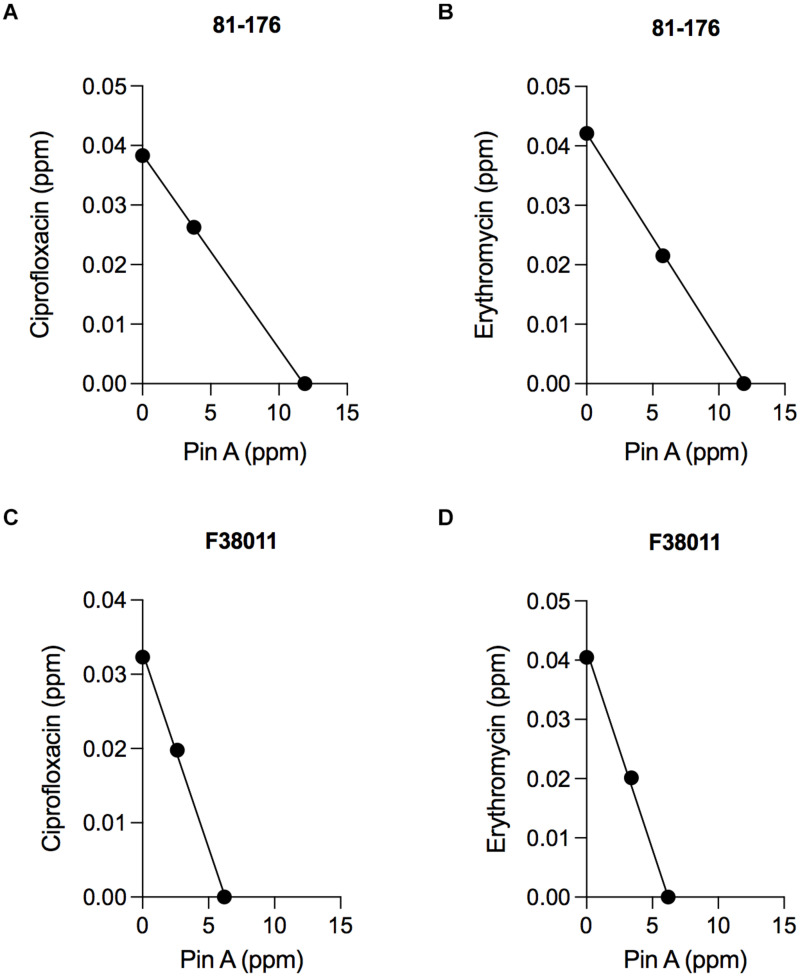
The combination effect of PinA with ciprofloxacin and PinA with erythromycin on *C. jejuni* strains. Isobologram analysis was used to investigate the interaction effect (synergistic, antagonistic or additive interaction) between binary combinations of PinA + ciprofloxacin **(A,C)** and PinA + erythromycin **(B,D)**. The additive line connects the IC_50_ of the two antimicrobial substances. Axial points show the IC_50_ of each antimicrobial treatment. An axial point on the line indicates additive interaction. An axial point above the additive line indicates an antagonistic interaction, whereas an axial point under the additive line indicates synergistic interaction. The assay was repeated a minimum of three times to ensure reproducibility.

Isobologram analysis was used to investigate the type of interaction effect (synergistic, antagonistic, or additive) between different binary combinations of PinA with ciprofloxacin and PinA with erythromycin. The IC_50_ of the antimicrobial agents was indicated on each axis, and the additive line was drawn by connecting the IC_50_ of the two antimicrobial substances. An axial point (combined effect) of the antimicrobial agents was then plotted. The type of interaction was then determined by the position of the axial point. An axial point on the line indicates an additive interaction, above the additive line indicates an antagonistic interaction, and under the additive line indicates synergistic interaction. Assays performed with *C. jejuni* strain 81-176 and the combination of PinA and ciprofloxacin (11.9 and 0.038 ppm, respectively) indicated an additive interaction, as did the combination of PinA and erythromycin (11.9 and 0.0415 ppm, respectively) (Figures 3A,B). Similar results were obtained for *C. jejuni* strain F38011 and the combination of PinA and ciprofloxacin (6.2 and 0.0323 ppm, respectively) and the combination of PinA and erythromycin (6.2 and 0.0405 ppm, respectively), where the combination of the two antimicrobial agents also indicated an additive interaction (Figures 3C,D). In summary, the combination of PinA with ciprofloxacin and PinA with erythromycin was identified to be more effective in reducing *C. jejuni* growth than using any of the antimicrobial agents alone.

### PinA Disrupts the *C. jejuni* Membrane

**FIGURE 4:**
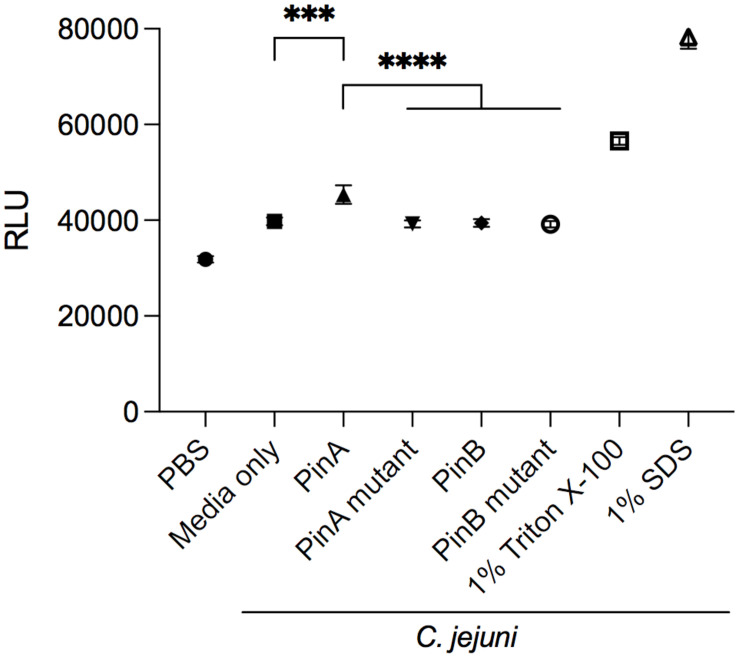
Effect of PinA and PinB peptides on cell membrane permeability of *C. jejuni* strain 81-176 cells. Ethidium bromide was added to *C. jejuni* strain 81-176 that were treated with PinA, PinA mutant, PinB, PinB mutant, 1% Triton X-100 or 1% SDS. Shown is the amount of accumulated ethidium bromide in untreated (no peptide) and peptide treated *C. jejuni* after a 40 min incubation period. The data represent three separate assays, and the error bars indicate the standard deviation from three assays. The asterisks indicate the relative fluorescence unit (RLU) for samples treated with PinA was statistically different than untreated cells (^∗∗∗^*P* < 0.001) and cells treated with PinA mutant, PinB, and PinB mutant peptides (^*⁣*⁣**^P < 0.0001), as judged by one-way ANOVA followed by Tukey’s multiple comparison test.

The mode of action of PinA and PinB has been studied previously. PinA was shown to disrupt membranes, probably due to its disulfide bond and hydrophobic TRD. The mode of action of PinB is different from PinA and likely targets intracellular components. To determine if PinA, PinB, or both peptides have the ability to disrupt the *C. jejuni* membrane, the ethidium bromide uptake assay was conducted. Ethidium bromide, a DNA intercalating agent, is known to be excluded from the intact cells and staining the DNA when cell membrane is damaged (Aeschbacher et al., 1986). The RLU for the bacterial cells treated with either PinA WT, PinA mutant, PinB WT, or PinB mutant peptides were obtained (Figure 4). As a negative control, PBS was added instead of peptide. One set of samples was left without the addition of any peptides or PBS (media only). As a positive control, 1% Triton X-100 and 1% SDS was used. As expected, the highest RLU was detected for the samples treated with 1% Triton X-100 and 1% SDS. Bacteria treated with PinA WT peptide showed a significantly higher (*P* < 0.001) RLU than the bacteria treated with PBS or only media. In contrast, no changes in the RLU were observed with bacteria treated with either the PinA mutant, PinB WT or PinB mutant peptides. Based on these results, PinA damages the *C. jejuni* cell membrane. However, PinB does not disrupt the *C. jejuni* membrane, suggesting some other modes of action.

### PinA Inhibits *C. jejuni* Metabolic Activity

**FIGURE 5:**
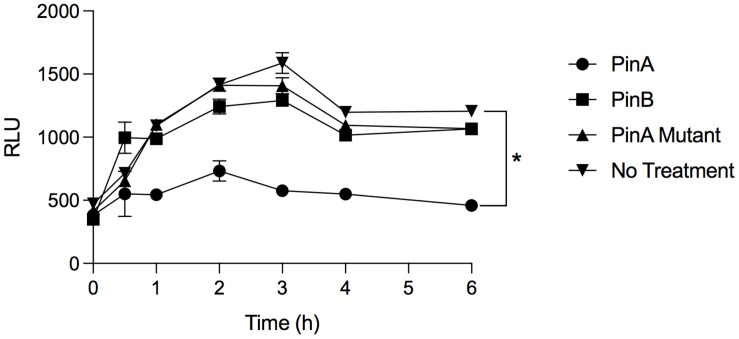
PinA inhibits *C. jejuni* metabolic activity. *C. jejuni* strain 81-176 was treated with PinA, PinB, PinA mutant peptides or left untreated in a 96-well plate and the bacterial metabolic activity was determined at different time points by ATP bioluminescence assay as described in section “Materials and Methods.” The data represent three technical replicates, and the error bars indicate mean ± standard error. The asterisk indicates that there is a statistically significant (*P < 0.05) differences in relative fluorescence unit (RLU) between the wells treated with PinA and no treatment control, as judged by one-way ANOVA followed by Dunnett’s multiple comparison test.

From our antimicrobial susceptibility test, we identified that PinA inhibited *C. jejuni* growth. However, it was not clear how quickly PinA inhibited the metabolic activity of bacteria, as the assays were performed after 48 h of incubation. To confirm the antimicrobial effect of PinA against *C. jejuni* strains and to determine the inhibition kinetics, bacterial metabolic activity was measured by the ATP bioluminescence assay. The ATP bioluminescence assay is a fluorometric assay that determines the amount of cytosolic ATP in biological samples. The greater the value, the higher the amount of ATP and the greater the metabolic activity. In this study, we identified that the PinA peptide inhibited the metabolic activity of *C. jejuni* strain 81-176 (Figure 5). At lower concentrations, the metabolic activity of the *C. jejuni* cultures increased quickly at early time points (Supplementary Figure 2A). However, the inhibitory effect of PinA at lower concentrations subsided after 6 h of incubation. Expectedly, PinB WT and PinA mutant peptides did not inhibit the metabolic activity of *C. jejuni* (Figure 5). There were no differences in *C. jejuni* metabolic pattern for PinB WT and PinA mutant at lower concentrations (Supplementary Figures 2B,C). For both PinB WT and PinA mutant peptides, the *C. jejuni* metabolic rate was increased at early time points (log phase) and then subsided at 4 h (stationary phase). However, metabolism was stable at 6 h, indicating the continuous metabolic activity of bacterial cultures. *C. jejuni* without any treatment (no peptides) showed a similar pattern of metabolic activity as seen with PinB WT and PinA mutant peptides suggesting no or minimal effect of PinB WT and PinA mutant peptides on *C. jejuni* growth. Overall, we identified that PinA effectively inhibits *C. jejuni* metabolic activity and that *C. jejuni* is unable to metabolize in the presence of a high concentration of PinA.

### Cytotoxic and Hemolytic Activity of PinA

**FIGURE 6:**
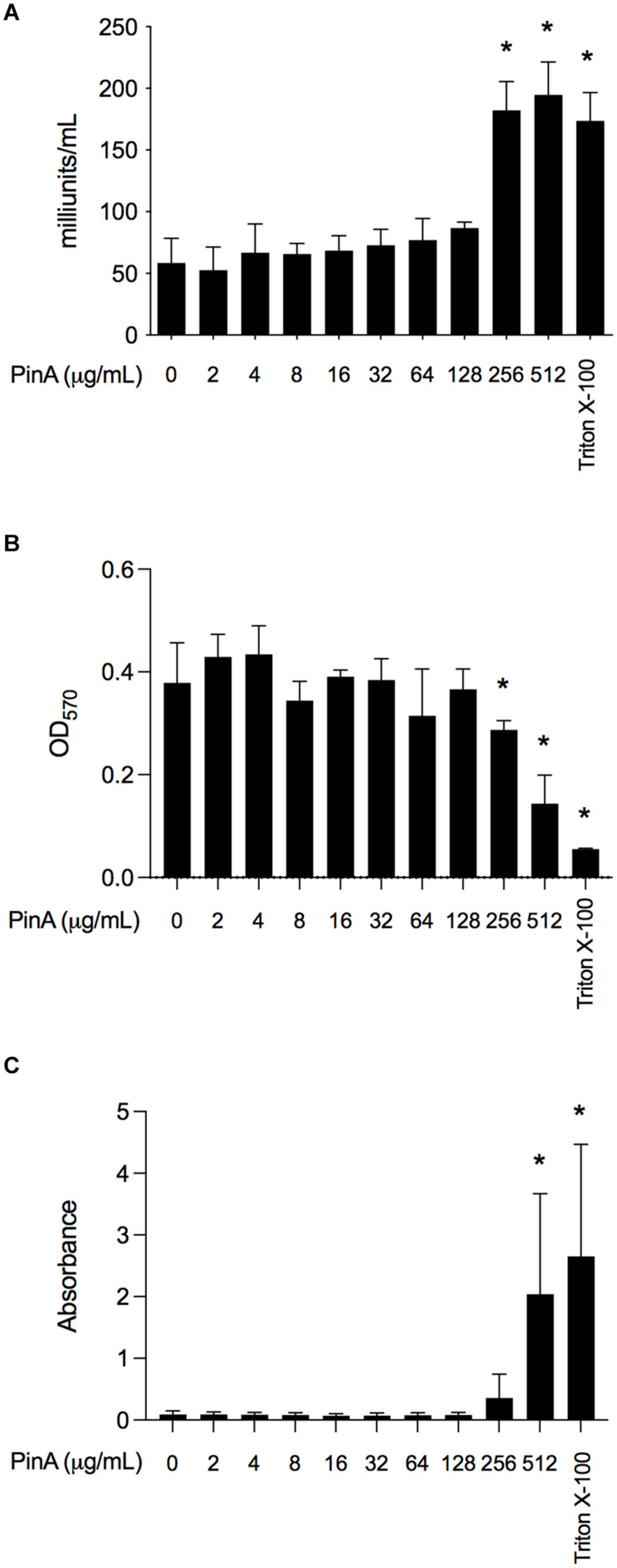
The cytotoxicity and hemolytic activity of PinA peptide. Cytotoxicity of PinA on INT 407 intestinal epithelial cells was obtained using the **(A)** lactate dehydrogenase (LDH) assay, and **(B)** MTT assay. Cells were treated with different concentrations of PinA, and the data (absorbance) were obtained for LDH and MTT assay as described in section “Materials and Methods”. **(C)** Hemolytic assay was performed with sheep red blood cells with different concentrations of PinA and the absorbance was obtained as described in section “Materials and Methods”. All assays were repeated at least three times, and the standard deviation is calculated from three separate assays. Statistical significance (*P < 0.05) of the values for each concentration compared to the untreated controls were calculated by one-way ANOVA followed by Dunnett’s multiple comparison test.

Next, we wanted to know whether PinA at inhibitory or bactericidal concentration could damage the host cells or exert any cytotoxic effect. The cytotoxicity of PinA and PinB peptides was tested against INT 407 cells (a derivative of HeLa cells) by two separate assays. LDH assay was used to measure the cell cytotoxicity, and MTT assay was used to measure the cell viability (Kumar et al.,2018a,b). INT 407 cells were treated with either the PinA WT or PinB WT peptides at different concentrations and incubated for up to 24 h at 37°C in a humidified condition. The reading was taken at the 3, 6, and 24 h time points. Different incubation times were used to determine the effect of short and long exposure of PinA and PinB peptides on INT 407 cells. The LDH assay results showed that PinA is cytotoxic to INT 407 cells at higher concentrations (Figure 6A). The cytotoxicity of PinA on INT 407 cells increased over time, as higher cytotoxicity was observed at 24 h compared to 3 and 6 h of incubation (data not shown). PinB did not show any cytotoxicity up to 512 μg/mL at any time point (Supplementary Figure 3A), suggesting that PinB is not cytotoxic to INT 407 cells. As a positive control for cytotoxicity, 0.1% Triton X-100 was used in this assay, and maximum damage was observed at all time points.

The MTT reagent determines the total ATP of the cell as the ATP content corresponds to the cell viability. The results showed that absorbance at OD_570_ is lower for PinA at a concentration of 256 μg/mL after 24 h, indicating cell death at high PinA concentrations (Figure 6B). However, no reduction of cell viability was observed for PinB at any concentration tested (up to 512 μg/mL) (Supplementary Figure 3B). A very low absorbance at OD_570_ was observed for cells treated with 0.1% Triton X-100, indicating minimum or no cell viability. In summary, the PinA peptide at higher concentrations is cytotoxic to INT 407 cells, whereas PinB does not have any cytotoxic effect on INT 407 cells at the highest concentrations tested (512 μg/mL).

Finally, we sought to determine the hemolytic activity of PinA WT, PinA mutant, PinB WT, and PinB mutant peptides with SRBCs. At a concentration of 256 μg/mL or higher, PinA showed hemolysis of SRBCs (Figure 6C). Similarly, the PinA mutant, PinB WT, and PinB mutant peptides also demonstrated hemolytic activity at high concentrations (256 μg/mL or higher) (data not shown). As positive and negative controls, Triton X-100 and PBS were used. In summary, our results indicate that PinA has hemolytic activity in mammalian cells at high concentrations.

### PinA Inhibits *C. jejuni* Biofilm Formation

**FIGURE 7:**
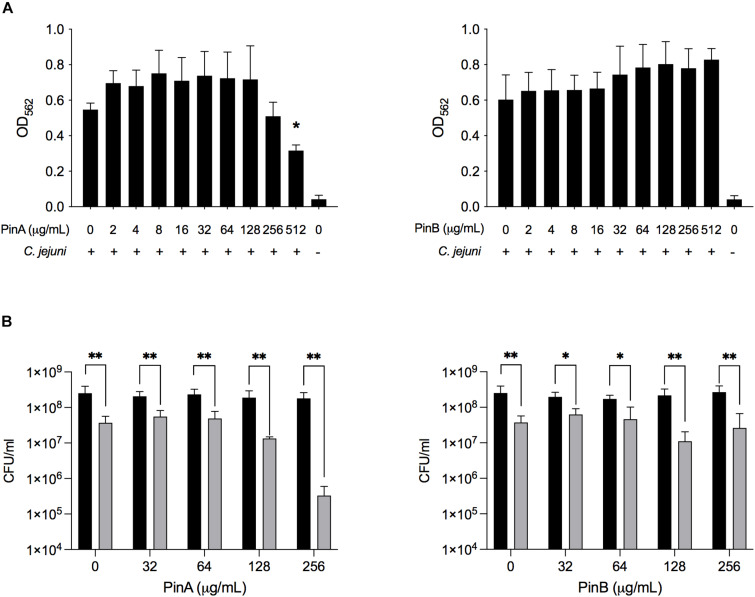
Effect of PinA and PinB peptides on *C. jejuni* biofilm formation and viability. *C. jejuni* biofilms were treated with the PinA and PinB peptides at different concentrations, and the effect of the peptides on **(A)** biofilm formation, and **(B)** bacterial viability was determined. The biofilm formation was determined by the crystal violet staining as described in section “Materials and Methods.” The number of live bacteria (CFU/mL) for planktonic (free living) (black bars) and sessile (biofilm associated) bacteria (gray bars) was counted by plating the culture on BHI agar plates with the appropriate dilution. The data represent three biological replicates and the error bars indicate the mean ± standard error. **(A)** The asterisks indicate that the biofilm formation by *C. jejuni* was statistically different (*P < 0.05) from untreated wells, as judged by one-way ANOVA followed by Dunnett’s multiple comparison test. **(B)** The asterisks indicate the significant difference (*P < 0.05, **P < 0.01) between the number of planktonic and sessile bacteria untreated or treated with different concentration of peptides, as judged by two-way ANOVA followed by Sidak’s multiple comparison test.

To evaluate the inhibitory effect of PinA and PinB on *C. jejuni* biofilm formation, different concentrations of the PinA WT and PinB WT peptides were used. Our preliminary results showed that at a concentration of OD_540_ of 0.05 or lower, the *C. jejuni* cultures did not produce a biofilm in 96-well tray after 72 h of incubation (as determined by the crystal violet assay, data not shown). Therefore, a higher density of *C. jejuni* cultures (OD_540_ = 0.1) was used for the biofilm assay. The biofilm assay coupled with crystal violet staining showed that there was inhibition in biofilm formation for *C. jejuni* cultures in a 96-well tray treated with PinA at a high concentration (Figure 7A). *C. jejuni* 81-176 formed significantly less biofilm with PinA at a concentration of 512 μg/mL (*P* < 0.05) than the untreated cultures (media only control). However, at low PinA concentrations (128 μg/mL or lower), the OD_562_ was similar or higher compared to the untreated cultures. When PinB was tested at concentrations up to 512 μg/mL in the biofilm assay, no significant inhibition (*P* > 0.05) of biofilm formation was observed (Figure 7A).

Next, the effect of PinA and PinB on planktonic and sessile bacteria was examined. In biofilms, planktonic bacteria are defined as the free-floating bacteria that can form a biofilm in a new system. Sessile bacteria are known as the bacteria associated with the biofilm itself. The liquid media and the adhered biofilm were serially diluted, plated and counted to determine the total number of planktonic and sessile bacteria in the wells (Figure 7B). The number of sessile bacteria in the biofilm decreased from 3.7 × 10^7^ CFU/mL with PinA at 0 μg/mL to 3.3 × 10^6^ CFU/mL with PinA at 256 μg/mL. The number of planktonic bacteria in the wells was between 1.8 × 10^8^ and 2.5 × 10^8^ CFU/mL with PinA at any concentration tested. The number of sessile bacteria was 6.2 × 10^7^ CFU/mL with PinB at 0 μg/mL and 1 × 10^7^ CFU/mL with PinB at 256 μg/mL. The number of planktonic bacteria in the biofilm wells was between 1.7 × 10^8^ and 2.7 × 10^8^ CFU/mL at any PinB concentration tested. However, the number of planktonic bacteria was significantly higher (*P* < 0.05) than the number of sessile bacteria at any given concentration tested for both PinA and PinB. In summary, our results indicate that the PinA peptide inhibits biofilm formation by *C. jejuni*, most likely by reducing the number of biofilm-associated sessile bacteria.

**FIGURE 8:**
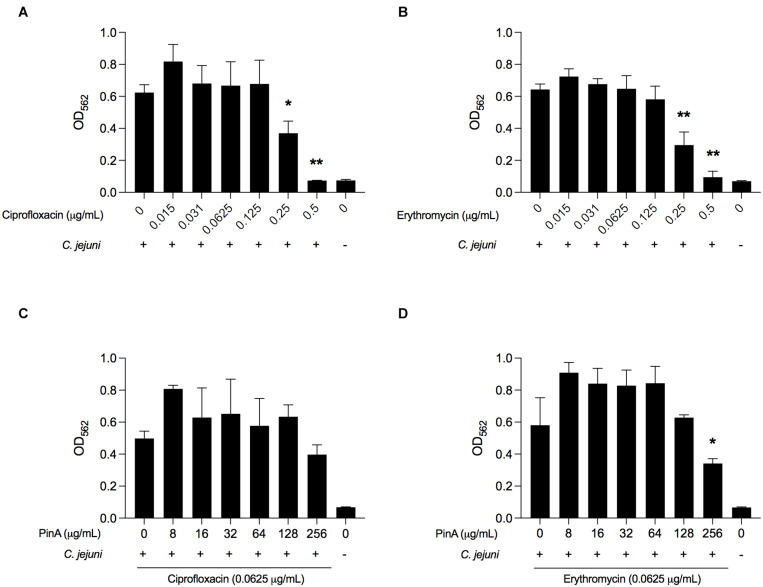
Effect of ciprofloxacin, erythromycin, and the combination of drugs on biofilm formation by *C. jejuni*. *C. jejuni* strain 81-176 was treated with **(A)** ciprofloxacin, **(B)** erythromycin, **(C)** ciprofloxacin and PinA, and **(D)** erythromycin and PinA in a 96-well tray and incubated at 37°C in a microaerobic condition for 72 h. The biofilm formation was determined by crystal violet staining as described in section “Materials and Methods.” The data represent three biological replicates, and the error bars indicate the mean ± standard error. The asterisks indicate that the biofilm formation by *C. jejuni* was statistically different (*P < 0.05, **P < 0.01) from untreated wells, as judged by one-way ANOVA followed by Dunnett’s multiple comparison test.

The isobologram analysis for biofilm formation was done to determine if there is any synergistic effect of using PinA in combination with either ciprofloxacin or erythromycin on inhibiting *C. jejuni* biofilm formation. Prior to applying the combination of PinA WT peptide with either ciprofloxacin or erythromycin in the biofilm assay, we tested the ability of *C. jejuni* to form biofilms in the presence of ciprofloxacin or erythromycin alone. Both ciprofloxacin and erythromycin inhibited *C. jejuni* biofilm formation at a concentration of 0.25 μg/mL or higher (Figures 8A,B). The checkerboard assay was then used to determine biofilm formation where different concentrations of the PinA peptide, ciprofloxacin, and erythromycin were used. In the checkerboard assay, PinA at a concentration of 256 μg/mL or higher inhibited the biofilm formation with all concentrations (0.125, 0.0625, 0.0312, and 0.0156 μg/mL) for both ciprofloxacin and erythromycin (Figures 8C,D). There was no difference in PinA activity at 256 μg/mL on *C. jejuni* biofilm formation between the regular biofilm assay and the checkerboard assay. This result suggests that there was no synergistic or additive effect of PinA with either ciprofloxacin or erythromycin, and that the PinA peptide alone can reduce biofilm formation at 256 μg/mL or a higher concentration.

## Discussion

The present study further establishes the antimicrobial role of the wheat puroindoline proteins against both Gram-positive and Gram-negative bacteria. In this study, we identified that the 18-mer synthetic PinA peptide containing the native TRD domain can inhibit bacterial growth *in vitro*. This finding is in support of the previous reports that showed the ability of full-length PinA protein or shorter PinA peptides harboring the TRD could inhibit the growth of different bacteria and fungi (Jing et al., 2003; Capparelli et al., 2005; Capparelli et al., 2006; Miao et al., 2012; Lv et al., 2019). However, unlike other studies, we did not observe antimicrobial activity with the PinB peptide at a concentration of 512 μg/mL or lower against any of the bacteria tested. To date, most investigators have found that PinA has a greater antimicrobial ability than PinB. However, two separate studies by Capparelli et al. (2005) and Capparelli et al. (2006) showed that PinB activity was much higher than PinA. The inactivity of PinB peptide in our study could be due to several reasons. First, in our study, we used the shorter 18-mer PinB peptide sequence instead of the full-length protein used in the studies of Capparelli and co-workers. Thus, the secondary structure of PinB used in this study may be different than the PinB peptides and proteins reported in other studies. Second, the lower solubility of PinB peptide in water or in MH broth may have resulted in the precipitation of peptide in the solution or the growth media. When the solubility of all synthetic peptides was tested, we observed that PinB peptide remains soluble in DMSO, an organic solvent, but not in water. In addition, the PinB peptide mixed with MH broth tended to precipitate during the incubation on a 96-well plate. Therefore, tested bacteria may escape the effect of PinB in liquid media due to the precipitation of the synthetic PinB peptides. The solubility of PinB with organic solvent is supported by the previous report showing that the puroindolines participate in both hydrophobic and ionic interactions, with a particular affinity for polar lipids (Morris et al., 1994; Greenblatt et al., 1995). Third, a unique set of bacterial isolates were used in this study. Although the PinB peptide was not observed to have antimicrobial activity against *C. jejuni* and other bacteria in this study, the antimicrobial role of PinB cannot be excluded until further studies are conducted with various PinB isoforms.

This study, in conjunction with previously published reports, has shown that the TRD is the key factor in determining the AMPs’ antimicrobial activity. It has been shown that changes or mutations in the TRD sequence abolish the antimicrobial role of these peptides. In contrast, PinA with an added second bioengineered TRD showed increased activity compared to the wild-type protein (Miao et al., 2012). In the present study, the W→G mutations in the TRD of PinA decreased PinA’s activity against all organisms. This decrease in activity is most likely due to a change in the peptide’s secondary structure and a lower affinity to the bacterial lipid membrane. In summary, the full-length PinA protein or shorter PinA peptide that harbors the TRD is effective against different organisms, including bacteria and fungi.

An important observation from this study is the non-additive role of PinA and PinB against *C. jejuni* growth. We identified that the PinA and PinB together act similarly as PinA alone against *C. jejuni*. Other studies have reported the cooperative role of PinA and PinB against different bacteria and fungi (Capparelli et al., 2005; Clifton et al., 2007). Another unique aspect of this study was to test the different conformation of PinA and PinB together. We used PinA and PinB peptides with two different combinations and joined by a glycine-rich linker sequences and tested whether either one of these conformations could have a similar inhibitory effect to PinA. When PinB is present at the N-terminal of PinA and joined by a glycine-rich linker sequence, it shows increased antimicrobial activity against *C. jejuni* strain 81-176 than PinB present at the C-terminal end of PinA (Supplementary Figure 1A). We hypothesize that PinB folded with PinA causing the blocking of TRD regions from exposure when it is present at the C-terminal end of PinA. Further studies of peptides having different conformations or with varying linker sequences will be helpful to understand the differences in antimicrobial properties of these peptides.

Although our major focus was to evaluate the antimicrobial effect of PinA and PinB peptides against two *C. jejuni* clinical strains, we tested other Gram-negative and Gram-positive bacterial isolates, including *E. coli* O157:H7, *S. aureus*, *S.* Typhimurium, and *L. monocytogenes*, primarily to compare our findings with previous reports (Capparelli et al., 2005; Capparelli et al., 2006). In addition, we wanted to confirm that the peptides that we designed and synthesized commercially were functional and can be used in future studies. Our study identified that PinA could inhibit the growth of all tested organisms, whereas the PinA mutant lost antimicrobial activity against those organisms. However, the MIC and MBC values of PinA were different for all bacterial isolates tested. Overall, the MIC values were higher compared to the MIC values reported in previous studies. For example, our MIC values for PinA were 64 μg/mL for *E. coli* and 128 μg/mL for MRSA. In previous studies, Capparelli and co-workers reported the MIC of PinA and PinB of 30 μg/mL for *E. coli*, *S. aureus*, and *S. epidermidis* when tested with both native and recombinant puroindoline proteins (Capparelli et al., 2005; Capparelli et al., 2006). In contrast, two other groups reported that the activity of PinA was higher than PinB against *E. coli* and *S. aureus* (Jing et al., 2003; Miao et al., 2012). The differences in the inhibitory effect of PinA and PinB peptides in the different studies are likely due to the variations in the synthesized proteins or peptides that lead to the change in solubility and conformation of the synthesized proteins or peptides. This phenomenon is supported by the study by Haney et al. (2013), where the authors synthesized a series of PinB variants with the addition of PinA sequence and identified that some of the PinB variants restored the lower antimicrobial activity of PinB.

Similar to the effect of growth inhibition, we also identified that PinA was able to inhibit the biofilm formation by *C. jejuni*. We identified that PinA was required at least 4× MIC concentration (>256 μg/mL) to inhibit the biofilm formation by *C. jejuni* strain 81-176. In two independent studies, Shagaghi et al. (2016, 2020) reported that PinA was required at a 2× MIC concentration to inhibit the biofilm formation by MRSA, *Pseudomonas aeruginosa*, *L. monocytogenes*, and *Listeria innocua*. In our study, the concentration of PinA peptide was higher (4× MIC) than studies by Shagaghi et al. (2016, 2020), as we used a higher bacterial inoculum (1 × 10^6^ bacteria/well) in the biofilm assay than in our antimicrobial susceptibility tests (inoculum size 5 × 10^4^ bacteria/well). The higher inoculum was used due to the inability of *C. jejuni* to form biofilm at lower cell densities. Bacteria form biofilms as a complex slime layer to protect themselves from external stresses, including antimicrobial agents. Many current antibiotics are unable to penetrate the bacterial biofilm layer and thus are deemed ineffective in inactivating bacteria inside the biofilm (Koo et al., 2017). Our findings suggest that PinA can penetrate the biofilm and inactivate sessile bacteria residing inside the biofilm. Therefore, PinA peptide can be used in addition to other natural products or traditional drugs as a potent anti-biofilm agent. However, further studies are needed to determine the effective concentration of PinA peptide with other biofilm-forming bacteria.

The cytotoxicity and hemolytic activity of PinA has been addressed in this study using human epithelial INT 407 cells and SRBCs. INT 407 cells were chosen for this study as these cells grow rapidly and easy to maintain in culture media. INT 407 cells have been used extensively in *C. jejuni* research for several decades (Negretti et al., 2019). While we found that PinA at higher concentrations is cytotoxic to both INT 407 cells and SRBCs, few previous studies reported different concentrations of PinA. The first report on the cytotoxicity of puroindolines was from Jing et al. (2003), where the authors used the blood hemolysis assay and identified no hemolytic activity for PinA and PinB at a concentration up to 1000 μg/mL. PinA also did not show any cytotoxicity against murine neuromuscular cells (Llanos et al., 2006) and *in vitro* hemolytic activity or toxicity on the murine macrophage J774 cells (Capparelli et al., 2007). In contrast to these earlier studies, a recent study showed that PinA is cytotoxic to HeLa cells (Shagaghi et al., 2020). In the present work, we also identified that PinA was cytotoxic against human INT 407 cells at higher concentrations. However, similar to previous studies (Jing et al., 2003; Capparelli et al., 2007), we did not observe any cytotoxicity of PinB at concentrations up to 512 μg/mL. We also observed that all puroindoline peptides, including PinA and PinB mutant peptides, have hemolytic activity at a high concentration (512 μg/mL or higher) with SRBCs. This is different from the observation by Capparelli et al. (2007), where they identified that PinA up to 150 μg/mL and PinB up to 50 μg/mL did not display hemolytic activity against red blood cells. We applied two individual assays to test the AMP’s cytotoxicity. LDH assay (measuring the LDH released from the damaged cells) was used to measure the cell cytotoxicity, and the MTT assay (determining the total amount of cytosolic mitochondrial dehydrogenase) was used to determine the cell viability. The two assays are complementary and confirm the cytotoxic effect of PinA at higher concentrations. Overall, PinA has antimicrobial activity against many organisms at a concentration that is lower than its cytotoxicity to mammalian cells. This result further strengthens the potential of PinA to be used as a therapeutic in clinical settings. However, future studies are needed to determine the cytotoxicity of PinA using an *in vivo* model.

Similar to earlier studies, this study also identified that PinA disrupts the *C. jejuni* membrane. By using scanning electron microscopy (SEM), Charnet et al. (2003) showed that PinA exerted its antimicrobial activity by forming pores in the lipid membrane of bacteria. In another study, Alfred et al. (2013) also used SEM with *Saccharomyces cerevisiae* treated with PinA-based peptide and showed the formation of pits or pores in cell membranes. Lv et al. (2019) suggested that the mechanism of PinA on fungi may be through cell wall destruction leading to the cell membrane, mitochondrial, and DNA damage, and eventually cell death. In the present study, we used the ethidium bromide uptake assay, which determines the presence of intracellular ethidium bromide by fluorescence. The increased amount of fluorescence indicates that more ethidium bromide was taken up, which corresponds to membrane disruption (Lin et al., 2002). We observed that PinA resulted in *C. jejuni* cell membrane disruption, causing the cell membrane to become permeable to an external component. However, the mechanism of PinB activity on *C. jejuni* was not tested as we did not see any antimicrobial activity of PinB in this study.

Overall, our study further strengthens the antimicrobial role of PinA against a broad range of microorganisms. Although this study mainly focuses on the foodborne pathogen *C. jejuni*, the findings of this study can be utilized in other organisms. The use of PinA with other antimicrobial agents or natural products could be used as potential therapeutic agents against bacterial infections. Puroindolines are located in the endosperm of wheat and are present in the end-products of wheat, such as flour and bread. Additionally, puroindolines are members of a larger family of “indolines” that are present in oat, barley, rye, and other commonly consumed cereals (Morris et al., 2021). Therefore, the consumption of puroindolines is considered safe and can be used as food preservatives. The potential of puroindolines also increases the use of other natural compounds as antimicrobials. The increased use of natural compounds as antimicrobial agents could be the key to mitigating the global problem of antibiotic resistance.

## Data Availability Statement

The raw data supporting the conclusions of this article will be made available by the authors, without undue reservation.

## Author Contributions

MK, CM, XL, and PT conceived the study and designed the experiments. PT, KT, TC, and MK performed the experiments. PT and KT analyzed the data. PT drafted the manuscript. MK, CM, and XL edited the manuscript. All authors contributed to the article and approved the submitted version.

## Conflict of Interest

The authors declare that the research was conducted in the absence of any commercial or financial relationships that could be construed as a potential conflict of interest.
