# Supplementary material for: Inhibitory Effect of Puroindoline Peptides on Campylobacter jejuni Growth and Biofilm Formation
Source: Front Microbiol. 2021 Jul 2;12:702762. doi: 10.3389/fmicb.2021.702762 (PMC8283790; doi:10.3389/fmicb.2021.702762)
Supplement: Supplementary file 1 [file Data_Sheet_1.docx]

**Inhibitory Effect of Puroindoline Peptides on *Campylobacter jejuni* Growth and Biofilm Formation**

Prabhat K. Talukdar^1^, Kyrah L. Turner^1^, Torin M. Crockett^1^, Xiaonan Lu^2^, Craig F. Morris^3*^, and Michael E. Konkel^1*^

^1^ School of Molecular Biosciences, College of Veterinary Medicine, Washington State University, Pullman, WA, United States

^2^ Department of Food Science and Agricultural Chemistry, Faculty of Agricultural and Environmental Sciences, McGill University, Montreal, QC, Canada

^3^ U.S. Department of Agriculture, Agricultural Research Service, Pullman, WA, United States

**SUPPLEMENTARY MATERIAL**

**Supplementary Table 1.** Antimicrobial susceptibility of PinA against different Gram-positive and Gram-negative microorganisms

| Organism | Concentration (µg/mL) | | | | | |
| --- | --- | --- | --- | --- | --- | --- |
|  | MIC^1^ | IC_90_^2^ | IC_50_^3^ | IC_10_^4^ | MBC^5^ |  |
| *Campylobacter jejuni* 81-176 | 32 | 27.7 | 11.9 | 3.8 | 64 |  |
| *Campylobacter jejuni* F38011 | 16 | 13 | 6.2 | 2.9 | 32 |  |
| *Escherichia coli* O157:H7 | 64 | 59.1 | 42.6 | 30.7 | 256 |  |
| *Salmonella enterica* serovar Typhimurium | 256 | 143.3 | 94.3 | 62.1 | 512 |  |
| *Staphylococcus aureus* | 128 | 80 | 50.8 | 32.2 | 256 |  |
| *Listeria monocytogenes* | 64 | 59.5 | 22.1 | 8.2 | 64 |  |

^1^ Minimum inhibitory concentration

^2^ 90% of maximal inhibitory concentration

^3^ Half maximal inhibitory concentration

^4^ 10% of maximal inhibitory concentration

^5^ Minimum bactericidal concentration

**Supplementary Table 2.** Antimicrobial susceptibility of ciprofloxacin and erythromycin against *Campylobacter jejuni* strains.

| Organism | Ciprofloxacin (µg/mL) | | | | |
| --- | --- | --- | --- | --- | --- |
|  | MIC^1^ | IC_90_^2^ | IC_50_^3^ | IC_10_^4^ | MBC^5^ |
| *C. jejuni* 81-176 | 0.09375 | 0.0489 | 0.0381 | 0.0297 | 0.09375 |
| *C. jejuni* F38011 | 0.0469 | 0.0449 | 0.0323 | 0.0232 | 0.09375 |
|  | Erythromycin (µg/mL) | | | | |
|  | MIC^1^ | IC_90_^2^ | IC_50_^3^ | IC_10_^4^ | MBC^5^ |
| *C. jejuni* 81-176 | 0.125 | 0.0677 | 0.0415 | 0.0254 | 0.125 |
| *C. jejuni* F38011 | 0.1875 | 0.0657 | 0.0405 | 0.0249 | 0.25 |

^1^ Minimum inhibitory concentration

^2^ 90% of maximal inhibitory concentration

^3^ Half maximal inhibitory concentration

^4^ 10% of maximal inhibitory concentration

^5^ Minimum bactericidal concentration

**Supplementary Figure 1.** Effect of PinA-linker-PinB, PinB-linker-PinA, and combination of PinA and PinB peptides on *Campylobacter jejuni* strains. *C. jejuni* strains (A) 81-176, and (B) F38011 were treated with peptides at different concentrations in a 96-well tray and incubated at 37 °C in a microaerobic condition. The values (OD_540_ nm) were taken after 48 hours of growth, and the % of bacterial inhibition was calculated by considering the values at 0 µg/mL peptides as 0% growth and bacteria only control as 100% growth (not shown). The data represent three technical replicates, and the error bars indicate mean ± standard error.

**Supplementary Figure 2.** Effect of PinA, PinB, and PinA mutant peptides on *Campylobacter jejuni* metabolic activity. *C. jejuni* strain 81-176 was treated with (A) PinA, (B) PinB, or (C) PinA mutant peptides at different concentrations in a 96-well tray, and the bacterial metabolic activity was determined at different time points by ATP bioluminescence assay as described in section “Materials and Methods”. The data represent three technical replicates, and the error bars indicate the mean ± standard error.

A

B

C

**Supplementary Figure 3.** Cytotoxic effect of PinB on intestinal epithelial cells. Shown are the data obtained from (A) lactate dehydrogenase (LDH) assay, and (B) MTT assay from INT 407 cells treated with different concentrations of PinB and incubated at 37 °C in a humidified condition (5% CO_2_) at 3 h, 6 h or 24 h. The assay was repeated at least three times to ensure reproducibility. Error bars indicate the mean ± standard error.

A

B
